# Normal Correction of Sodium Leading to Central Pontine Demyelinosis: A Rare Occurrence

**DOI:** 10.7759/cureus.3252

**Published:** 2018-09-04

**Authors:** Fasih Sami Siddiqui, Zarmina Javed, Ujala Mahmood, Izza Saeed, Yusaf F Qasim, Muhammad Saim Bin Saeed

**Affiliations:** 1 Internal Medicine, Shifa College of Medicine, Islamabad, PAK; 2 Internal Medicine, Shifa College of Medicine/Shifa International Hospital, Islamabad, PAK; 3 Medicine, Shifa International Hospital, Islamabad, PAK; 4 Internal Medicine, Shifa College of Medicine, Rawalpindi, PAK

**Keywords:** normal sodium correction, cpm, central pontine myelinolysis

## Abstract

Central pontine myelinolysis is a rare condition arising from the myelinolysis of white matter tracts in the pons, most commonly in response to iatrogenic hypertonic stress caused by the rapid correction of hyponatremia. Here, we present an interesting case of central pontine myelinolysis subsequent to normal saline infusion despite strict adherence to the guideline protocol.

## Introduction

Central pontine myelinolysis (CPM) is a rare condition arising from the myelinolysis of white matter tracts in the pons, most commonly in response to iatrogenic hypertonic stress caused by the rapid correction of hyponatremia [1]; however, the disorder has also infrequently arisen in other clinical settings [2]. The classic clinical course is biphasic, with a patient developing the complications of hyponatremia (e.g. seizures, encephalopathy) that subside quickly after rectifying serum sodium levels only to degenerate two to eight days later [3]. Here, we present an even more uncommon case where CPM occurred despite strict adherence to the guideline protocol.

## Case presentation

A 50-year-old male with a known history of coronary artery disease (CAD), hypertension, and alcohol abuse was brought into the emergency department (ED) in an obtunded state by emergency medical services. En route to the hospital, he had an episode of a generalized tonic-clonic seizure (GTCS), which subsided with the administration of intravenous (IV) midazolam.

Initial presentation to the ED revealed a patient with fluctuating consciousness and a Glasgow Coma Scale (GCS) score of 11/15. Vitals included a temperature of 36.6 C, a blood pressure of 178/107 mm Hg, a heart rate of 92 beats per minute, oxygen saturation of 90%, and a respiratory rate of 20 breaths per minute. The physical examination was unremarkable while a neurologic exam was limited due to sedation. Cough and gag reflexes were intact.

Initial laboratory investigations are presented in Table 1.

**Table 1:** Laboratory values on admission BUN: blood urea nitrogen; ALT: alanine aminotransferase; AST: aspartate aminotransferase; ALP: alkaline phosphatase

Serum sodium	101 mEq/L
Serum potassium	4 mEq/L
Serum chloride	78 mEq/L
Serum bicarbonate	23 mEq/L
BUN	4 mg/dL
Serum creatinine	0.55 mg/dL
Random blood glucose	135 mg/dL
Total protein	7.4 g/dL
Serum albumin	3.8 g/dL
ALT	90 U/L
AST	136 U/L
ALP	153 U/L
Total bilirubin	1.7 mg/dL
Direct bilirubin	0.5 mg/dL
Indirect bilirubin	1.2 mg/dL

Considering a low serum osmolality of 231 mEq/L, a low random urine osmolality of 161 mOsm/kilogram of water, and a lack of prior medications that could be attributed to the decreasing levels of serum sodium, his hypovolemic hyponatremia was attributed to psychogenic polydipsia and/or poor oral solute intake. The patient was initially treated with an infusion of two liters of normal saline (NS) and 200 mL of hypertonic saline. The serum sodium rose to 112 mEq/L with this infusion in the first 24 hours. An appropriate rise in serum sodium was observed in the ensuing four days, at a rate of 8 mEq per 24 hours. During the course of his medical intensive care unit (MICU) stay, the patient remained somnolent and visibly encephalopathic, which led to a self-extubation on the fifth day of his admission. He maintained a near-normal oxygen saturation on room air and was, therefore, continued on oxygen via a nasal cannula.

**Figure 1:**
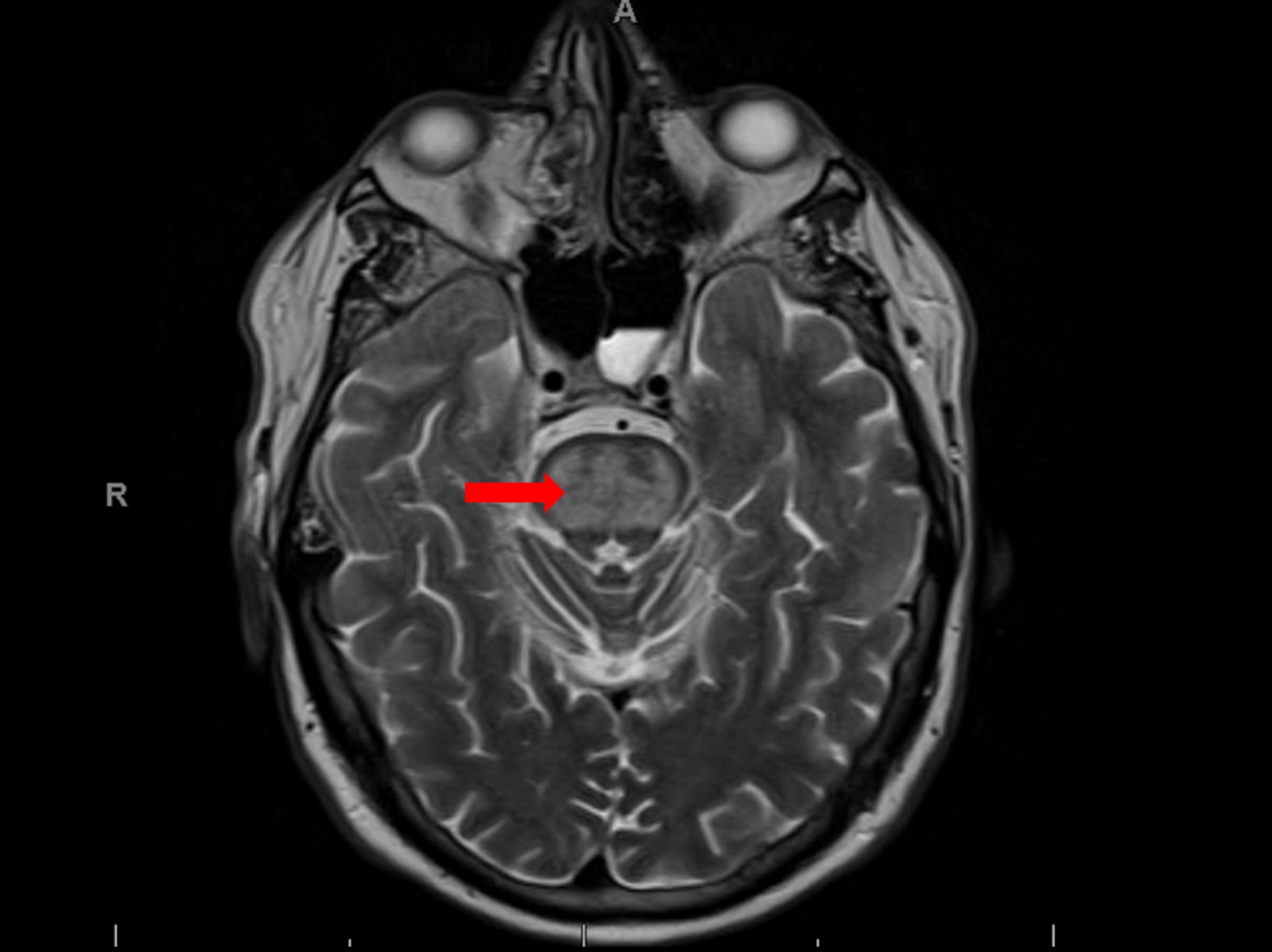
Magnetic resonance imaging revealed edema and swelling in the pons (red arrow) consistent with pontine demyelinosis

The patient’s altered mentation was initially attributed to multifactorial etiologies, which included intensive care unit (ICU) delirium, acute hypoxic encephalopathy, and electrolyte abnormalities. An appropriate sodium correction and a lack of hypoxic episodes prior to or after self-extubation ruled out the differentials, explaining his altered mentation. Despite adequate treatment, a persistent somnolence prompted a brain magnetic resonance imaging (MRI) scan whose findings were consistent with osmotic pontine demyelination. Imaging revealed edema and swelling in the pons, which was consistent with a subacute injury related to osmotic demyelination. Edema involving the internal/external capsules, thalamus, and amygdala as well as enhancement involving the subcortical white matter in the temporal lobes were also observed and attributed to a likely related subacute osmotic injury (Figure 1).

The patient continued to have difficulty in efficiently managing his oral secretions secondary to his neurological deficits and eventually aspirated with resultant findings of aspiration pneumonia in the ICU setting. He was treated with ampicillin-sulbactam for the duration of this predicament, five liters of oxygen via nasal cannula and frequent oral/pharyngeal suction. A subsequent neurology and palliative care consult concluded that the best approach moving forward was for the patient to undergo aggressive physical, occupational, and speech therapies over the next eight weeks and to observe for signs of improvement. The patient is currently followed in the outpatient department (OPD) with frequent follow-ups while further decisions regarding goals of care will be made after the resolution of these intensive therapeutic strategies.

## Discussion

Osmotic demyelination syndrome (ODS) is an acute neurological disorder caused by damage to the myelin sheath of neurons [1]. Due to the greater susceptibility of the pons, the central pontine myelinolysis (CPM) subtype is the classical presentation. CPM was initially described in 1959 as a new disorder in a report of four patients who were chronic alcoholics and malnourished [2]. Early authors concentrated on alcoholism and it was not until the late 1970s that the connection with electrolyte abnormalities was established [3].

Although the precise pathogenesis remains unclear, the most frequent trigger of myelinolysis in clinical practice is the rapid correction of chronic hyponatremia [2]. Osmotic demyelination has also been observed in other settings in the absence of rapid serum correction, such as hyperosmolar hyperglycemia, hyperammonemia, hypoxia, severe liver disease, and chronic alcoholism [4]. It is postulated that the brain adapts to chronically low levels of the intracellular osmolyte, and the subsequent iatrogenic hypertonic stress owing to rapid correction of hyponatremia causes the ions and water to quickly re-enter the intracellular space. As this occurs, the intracellular sodium and chloride levels rise to a higher than normal value, resulting in cellular dehydration [5].

Recent guidelines from the United States suggest that correction rates for hyponatremia not exceed 8 mEq/L for any 24-hour period in patients who carry a high risk for osmotic demyelination (e.g., serum sodium <=105 mEq/L, hypokalemia, malnutrition, or liver disease). European guidelines propose limiting correction to <=10 mEq/L in the first 24 hours and <=8 mEq/L for any 24-hour period thereafter [6]. However, there have been cases such as ours, although rare, where CPM occurred despite the fact that guideline instructions were implemented absolutely. The current standard method for the diagnosis of CPM is brain magnetic resonance imaging (MRI). T1-weighted images will show the symmetric hypointense lesions; meanwhile, T2-weighted images have symmetric hyperintense lesions [7-8].

In literature, several treatment modalities, such as thyrotropin-releasing hormone, plasmapheresis, steroids, and immunoglobulins have been proposed for CPM but due to a lack of randomized controlled trials, they have not been included in the treatment guidelines. To date, the most prominent approach to CPM is supportive management and preventing secondary life-threatening complications [9].

The prognosis of CPM varies widely from almost complete recovery to partial or no recovery at all. Although previously thought to be almost consistently fatal or severely debilitating, CPM is actually compatible with good recovery in the majority of cases [2]. The prompt diagnosis of CPM with MRI and the prompt initiation of treatment could explain this decline in overall mortality [5]. Some of the favorable outcomes for CPM include in the presence of higher Glasgow Coma Scale (GCS) scores, better scores on functional scales used in hospitals at admission, discharge and last follow-up, less severe hyponatremia, and absence of superadded hypokalaemia [10].

## Conclusions

Central pontine myelinolysis (CPM) is a neurological disorder infrequently encountered in the clinical setting. Most commonly, it is precipitated by iatrogenic hypertonic stress from rapid infusion, leading to the myelinolysis of white matter tracts in the pons. Although rare, patients with a slow correction of hyponatremia may also develop CPM. Contrary to previously held beliefs, patients with CPM often achieve favorable recovery but a paucity of randomized control trials means that the treatment of CPM is restricted to providing supportive care and preventing further complications.

## References

[1] Brown WD (2000). Osmotic demyelination disorders: central pontine and extrapontine myelinolysis. Curr Opin Neurol.

[2] Singh TD, Fugate JE, Rabinstein AA (2014). Central pontine and extrapontine myelinolysis: a systematic review. Eur J Neurol.

[3] Alleman AM (2014). Osmotic demyelination syndrome: central pontine myelinolysis and extrapontine myelinolysis. Semin Ultrasound CT MR.

[4] George JC, Zafar W, Bucaloiu ID, Chang AR (2018). Risk factors and outcomes of rapid correction of severe hyponatremia. Clin J Am Soc Nephrol.

[5] Sheikh A, Afzal RM, Sagheer S (2018). The dilemma of inadvertent pontine demyelinosis: a review of literature. Cureus.

[6] Hoorn EJ, Zietse R (2017). Diagnosis and treatment of hyponatremia: compilation of the guidelines. J Am Soc Nephrol.

[7] Chua GC, Sitoh YY, Lim CC, Chua HC, Ng PY (2002). MRI findings in osmotic myelinolysis. Clin Radiol.

[8] Hurley RA, Filley CM, Taber KH. (2011). Central pontine myelinolysis: a metabolic disorder of myelin. J Neuropsychiatry Clin Neurosci.

[9] Musana AK, Yale SH (2005). Central pontine myelinolysis: a metabolic disorder of myelin. WMJ.

[10] Kallakatta RN, Radhakrishnan A, Fayaz RK, Unnikrishnan JP, Kesavadas C, Sarma SP (2011). Clinical and functional outcome and factors predicting prognosis in osmotic demyelination syndrome (central pontine and/or extrapontine myelinolysis) in 25 patients. J Neurol Neurosurg Psychiatry.

